# Oral Hygiene Practices and Associated Factors among Patients Visiting Private Dental Clinics at Hawassa City, Southern Ethiopia, 2018

**DOI:** 10.1155/2021/8868308

**Published:** 2021-03-26

**Authors:** Desalegn Humna Beyene, Bereket Beyene Shashamo, Lankamo Ena Digesa, Eshetu Zerihun Tariku

**Affiliations:** ^1^Bon Dental Clinic PLC, Addis Ababa, Ethiopia; ^2^School of Nursing, Arba Minch University, Arba Minch, Ethiopia; ^3^School of Public Health, Arba Minch University, Arba Minch, Ethiopia

## Abstract

**Introduction:**

A poor oral hygiene is associated with dental caries, gingivitis, periodontal diseases, bad breath, respiratory and cardiovascular diseases, and chronic kidney diseases. Moreover, a poor oral health has psychosocial impacts that diminish a quality of life and restrict activities in school, at work, and home. African regions carry a major burden of oral health problems. However, very few studies highlighted about oral hygiene practices and there is also paucity of information in Ethiopia. This study was, therefore, designed to identify an oral hygiene practice on patients/clients visiting dental clinics in Hawassa City, Southern Ethiopia.

**Objective:**

To assess oral hygiene practices and associated factors among patients/clients visiting private dental clinics, Hawassa City, Southern Ethiopia.

**Methods:**

Institution-based cross-sectional study was employed among patients/clients attending private clinics in Hawassa City from January 27 to February 8, 2018. Systematic random sampling technique was used to select 403 study participants. Data were entered into EpiData 3.1, cleaned, and analyzed by SPSS 20. A multivariable logistic regression analysis was performed to assess the association between independent and outcome variables. Crude and adjusted OR with 95% confidence level was estimated, and variables having *P* value ≤0.05 in multivariable analysis were considered as significant.

**Results:**

393 study participants participated making a response rate of 97.52%. A median age of respondents was 27 ± 10.9. About 153 (39.9%) of the study participants had poor oral hygienic practice. Male (AOR: 1.63, 95% CI: (1.053, 2.523)), rural residence (AOR: 3.79, 95% CI: (1.724, 8.317)), and poor knowledge about oral hygiene (AOR: 2.38, 95% CI: (1.402, 4.024)) were independently associated to poor oral hygienic practice.

**Conclusion:**

More than one-third of the study participants had poor oral hygienic practice. Providing health information regarding oral hygiene for the patients/clients in the facilities with a special focus from rural areas is recommended.

## 1. Introduction

An oral hygiene is maintaining cleanness of mouth and taking care of teeth and gum [1–4]. A good oral hygiene practice promotes better oral health and general health of person [5–7]. A poor oral hygiene is associated with dental caries, gingivitis, periodontal diseases, bad breath, respiratory diseases, cardiovascular diseases, and chronic kidney diseases [8–10]. Moreover, a poor oral health has a psychosocial impact that diminishes quality of life and restricts activities in school and at work and home [8, 10].

Periodontal disease and dental caries are the major problems of the world [11]. About 3.9 billion people of the world were affected by burdens of oral conditions [12]. The study in India [13] depicted that the knowledge regarding oral health was low. Another study in Qatar [2] revealed that 40% of the study respondents were found having poor oral status. Similarly, the study in Pakistan showed that practice of respondents about oral hygiene was poor [14].

Oral diseases are the major public health problem of African region [11, 15]. African regions carry a major burden of oral health problems because of this oral disease is one of the prioritized noncommunicable diseases [16]. The burden of an oral disease in low economic communities is noticeable [11]. The challenges of responding to an oral health are prominent in developing communities [12]. A disability and death related with a poor oral condition in East Sub-Saharan Africa was significantly higher than other Sub-Saharan African countries [12]. A study in Nigeria [17] revealed that there was a poor oral health knowledge among adult Nigerians.

Rural residence and poor knowledge were associated with poor oral hygiene practice [1–4]. On the other, the study in Qatar [2], Eastern India [3], and Addis Ababa [4] revealed that being female, urban residence, educational status secondary and above, and adequate knowledge were associated with better oral hygiene practice.

The study in Addis Ababa [18] reported that the prevalence of periodontal diseases and dental caries was high 35.4% and 47.4%, respectively. About 87.7% of the respondents clean their teeth once or more in a day and only 23.2% respondents reported as they floss their teeth [4]. Moreover, the studies in Addis Ababa [4, 18] depicted that the practice of tooth brushing was low. There are a few studies in Ethiopia that address about an oral hygiene practice with a limited geographic coverage. This study was, therefore, designed to identify an oral hygiene practice among patients/clients visiting private dental clinics in Hawassa City, Southern Ethiopia.

## 2. Methods and Materials

### 2.1. Study Area and Period

The study was conducted in Hawassa City, Southern Ethiopia from January 27 to February 8, 2018. Hawassa is located 273 km to the South of Addis Ababa, the capital of Ethiopia. Based on the 2007 Census conducted by the Central Statistical Agency of Ethiopia, the city has a total population of 258,808 [19]. Dental service in the city is provided by one governmental hospital (Hawassa University Referral Hospital) and 8 private dental clinics. The private clinics were selected because most of dental services in Ethiopia are provided in private dental clinics.

### 2.2. Study Design and Participants

Institution-based quantitative cross-sectional study design was employed. Patients/clients visiting private dental clinics in Hawassa City during the data collection period and age of 15 years and above were included, and those patients who were seriously ill were excluded from the study.

### 2.3. Sample Size and Sampling Procedure

Sample size for this study was calculated using a single population proportion formula with the assumptions of proportion of poor oral hygiene practice (50%) among patients, 95% confidence interval, 5% margin of error, and 5% nonresponse rate. Accordingly, the total sample size for this study was 403.

Systematic sampling technique was employed to select study participants from all [8] private dental clinics in the city, and the sample size was proportional allocated to each dental clinic. From the pre-data collection assessment, in an average, 140 clients/patients visit each clinic within 2 weeks. Accordingly, 50 clients/patients were selected from each clinic using systematic sampling technique. The first participant was selected using the lottery method, and the next was selected every 3 clients/patients until the required sample size was fulfilled.

### 2.4. Operational Definitions

- Good knowledge: corresponds to a score of mean and above for 9 knowledge questions [20]. The mean score for the correct response was 6 for this study.
- Poor knowledge: corresponds to a score of below mean for 9 knowledge questions [20]. The mean score for the correct response was 6 for this study.
- Good oral hygiene practice: corresponds to a score of mean and above for 11 practice questions [20]. And the mean score for the correct response was 7 for this study.
- Poor oral hygienic practice: corresponds to a score of below mean for 11 practice questions [20]. And the mean score for the correct response was 7 for this study.

### 2.5. Data Collection Tool and Procedure

Data were collected using a structured interviewer-administered Amharic version questionnaire. The questions were adopted from studies that were conducted in Addis Ababa, Ethiopia [4, 18]. The face-to-face interview was done at a separate room after the client/patient received the service. Six diploma nurses and two BSc nurses were recruited as a data collector, and two BSc nurses supervised the data collection. The data collection tool comprises three parts: questions for assessing sociodemographic attributes, questions for assessing knowledge level of the patients regarding oral hygiene, and questions designed to assess practice of oral hygiene practice.

### 2.6. Data Quality Control

The questionnaire that was initially prepared in English language was translated to Amharic language (the local language in the study area) and back translated to English by language experts for consistency. The questionnaire was pretested in 5% of the sample before the actual data collection period. Necessary amendments were done based on the findings of the pre-test, and amended tool was used for the actual data collection. Training was given for data collectors and supervisors on objectives of the study, questionnaires administration, and the data collection procedure.

### 2.7. Data Processing and Analysis

The collected data were coded and entered into EpiData version 3.1. Then, the data were exported, cleaned, and analyzed by SPSS20. Descriptive analyses such as simple frequencies, measures of central tendency, and measures of variability were used to describe the characteristics of participants. Then, the information was presented using frequencies, summary measures, and tables.

Binary logistic regression was employed to assess the association between independent and outcome variables. The variables with *P* value ≤0.25 in bivariate analyses were entered into multivariable logistic regression to control confounders. Crude and adjusted odds ratios with 95% CI were estimated to identify the factors associated with patients' oral hygiene practices using multivariable logistic regression analyses. Level of statistical significance was declared at *P* value ≤0.05. Model fitness was checked using the Hosmer–Lemshow test.

### 2.8. Ethical Consideration

Formal letter was obtained from Atlas Health Sciences College. Data were collected after formal permission letter was obtained from Hawassa City Health Bureau to all included private dental clinics. The purpose of the study was clarified to each study participant before beginning of data collection. A written informed consent was taken. For those who were under 16 years of age, a written guardian or parent consent was obtained. Only those who gave consent to participate in the study were included in the study. The study participant was coded without name; confidentiality of information was maintained.

## 3. Results

### 3.1. Sociodemographic Characteristics of the Study Participants

393 study respondents participated making a response rate of 97.52%. From the respondents, 383 (97.45%) of them responded that they clean their teeth. From the study participants those who clean their teeth 204 (53.3%) were males. About 359 (93.7%) were 18 and above years old with median 27 (SD ± 10.9). Majority of the participants 335 (87.5%) had secondary and above educational level. Three hundred forty-five (90.1%) of the respondents are living in urban area (Table 1).

### 3.2. Patients/Clients' Practices Regarding Oral Hygiene

383 (97.46%) of them reported that they clean their teeth. From the study respondents, the majority (53.8%) of them clean their teeth regularly and 294 (76.8%) of them use toothpaste to clean their teeth. Only 31 (8.1%) of them rinse their teeth after eating (Table 2).

### 3.3. The Overall Level of Oral Hygiene Practices among Patients

About 153 (39.9%) of the patients had poor practice regarding oral hygiene.

### 3.4. Patients' Knowledge Regarding Oral Hygiene

Majority (80.4%) of the participants do not know that cleaning their teeth brightens their teeth but 322 (84.1%) of them knew that sweet foods can affect teeth adversely (Table 3).

### 3.5. Overall Knowledge Level Regarding Oral Hygiene among Patients

About 104 (27.2%) of the study participants had poor knowledge regarding oral hygiene.

### 3.6. Factors Associated with Oral Hygiene Practice among Patients

In multivariable analyses, sex of respondents, residence, and knowledge regarding oral hygiene were identified to be significantly associated with oral hygiene practices of the patients (Table 4).

Male patients were 1.6 times (AOR = 1.63, 95% CI: (1.053, 2.523)) more likely to have poor practice regarding oral hygiene when compared to female patients. Patients who live in rural area were 3.8 times (AOR = 3.79, 95% CI: (1.724, 8.317)) more likely to have poor practice regarding oral hygiene when compared to the patients who live in urban area. The odds of having poor oral hygiene practice were 2.4 times (AOR = 2.38, 95% CI: (1.402, 4.024)) higher among patients who had poor knowledge regarding oral hygiene when compared to patients who had good knowledge.

## 4. Discussion

In the present study, more than one-third of the patients had poor practice regarding oral hygiene. Among the factors, being male, living in rural area, and having poor knowledge regarding oral hygiene were significantly and independently associated with poor oral hygiene practice. The factors independently associated with poor oral hygiene practice will be discussed as the follows with 95% confidence intervals.

This study has revealed that poor oral hygiene practice was almost two times more common among males than their females' counterpart. This finding is in conformity with the finding from a study conducted in India [3, 21], where females had a better oral hygiene practice than the males. This is because women are more conscious about their body image and appearance than males [22]. However, it is in contrary with study finding from Qatar which showed that women had poor practice of oral hygiene [2]. This discrepancy could be due to difference in cultural background of the study respondents, i.e., from Ethiopia and Qatar.

This study depicted that the rural residents are nearly 4 times more likely to practice oral hygiene poorly when compared to urban residents. This is in line with the finding from the study conducted in India [21].

The current study showed that participants who have poor knowledge about oral hygiene were nearly two times more likely to have poor practice of oral hygiene than those who have good knowledge. This is in agreement with the finding in Addis Ababa, Ethiopia [4]. This could be explained by the possibility that those patients/clients who have a better knowledge about oral hygiene are also at a better position to have good oral hygienic practice.

According to the finding of this study, majority of the respondents clean their teeth once a day. However, the finding in Addis Ababa revealed that nearly one-fourth of the respondents clean their teeth [4].

## 5. Conclusion and Recommendations

The present study found that the patient's practice regarding oral hygiene was poor, in which more than one-third of the participants had poor oral hygiene practice. This study also revealed that males, rural residents, and the participants with a poor knowledge were associated with poor oral hygiene practice. Providing health information, in collaboration with corresponding stakeholders, regarding oral hygiene is recommended for those patients who come to visit dental clinics. The messages should focus on the frequency, timing, and techniques of tooth brushing with major emphasis for those patients coming from the rural areas. The participants in this study were from private dental clinics, and the findings may have great implications regarding the oral health in the community of the study area. Many patients/clients visiting private dental clinics are from a richer family. Those clients who do not visit the clinics because of low economic status may visit traditional healers and they will be at a greater extent of suffering from the consequences of poor oral hygiene. Therefore, a community level education to enhance public awareness about oral health is necessary. Since clients who visit private clinics are more likely from higher economics status, this may underestimate the prevalence of poor oral hygiene.

## Figures and Tables

**Table 1:** Sociodemographic characteristics of the patients/clients visiting private dental clinics, Hawassa City, Southern Ethiopia, 2018 (*n* = 383).

Variables	Category	Frequency	Percent
Sex of participants	Male	204	53.3
	Female	179	46.7
Age in years	<18 years	24	6.3
	≥18 years	359	93.7
Educational level	Below secondary	48	12.5
	Secondary and above	335	87.5
Average monthly household income in ETB	<5000	174	45.4
	≥5000	209	54.6
Employment status	Yes	218	56.9
	No	165	43.1
Residence	Rural	38	9.9
	Urban	345	90.1

**Table 2:** Frequency and percentage distribution of patients' practices regarding oral hygiene in dental clinics, Hawassa City Administration, Southern Ethiopia, 2018 (*n* = 393).

Items	Category	Frequency	Percent
Cleaning teeth	Yes	383	97.5
	No	10	2.5
Frequency of cleaning teeth	Regularly	206	53.8
	Sometimes	177	46.2
Cleaning aid used	Tooth brush	294	76.8
	Finger	6	1.6
	Tree stick	77	20.1
	Others	6	1.6
Cleaning material	Tooth paste	294	76.8
	Tooth powder	5	1.3
	Charcoal	21	5.5
	Others	63	16.4
Frequency of cleaning teeth per day	Once a day	228	59.5
	Twice a day	127	33.2
	More than twice daily	28	7.3
Aids to clean interdental areas	Never	211	55.1
	Sometimes	172	44.9
Frequency of changing tooth brush	<2 months	164	42.8
	2-3 months	117	30.5
	3 up to 6 months	70	18.3
	>6 months	32	8.4
Time of cleaning teeth	Less than 3 minutes	197	51.4
	3 minutes or above	186	48.6
Tooth paste used	Fluoridated tooth paste	96	25.1
	Nonfluoridated	58	15.1
	Do not know	229	59.8
Technique of tooth brushing	Circular	42	11
	Horizontal	138	36
	Vertical	56	14.6
	Combined	147	38.4
Cleaning tongue	Yes	251	65.5
	No	132	34.5
Rinsing mouth after eating	Yes	352	91.9
	No	31	8.1

**Table 3:** Frequency and percentage distribution of patients' knowledge regarding oral hygiene in dental clinics, Hawassa City Administration, Southern Ethiopia, 2018 (*n* = 383).

Variable	Category	Frequency	Percent
Source of information about oral health	Radio and television	222	58
	Friends	39	10.2
	Community	75	19.6
	Newspaper	47	12.3
Cleaning your teeth brightens teeth	Yes	75	19.6
	No	308	80.4
Cleaning teeth prevents gum bleeding	Yes	41	10.7
	No	342	89.3
Cleaning teeth prevents oral cancer	Yes	22	5.7
	No	361	94.3
Cleaning teeth prevents foul breath	Yes	245	64
	No	138	34
Action during gums bleeding	Stop brushing	31	8.1
	Go to see a dentist	242	63.2
	Brush more frequently	56	14.6
	Pay more attention when brushing	26	6.8
	Never had this problem before	28	7.3
Action during seeing a sign of decay	Just try to cope with the problem	11	2.9
	Do not care if no pain	77	20.1
	Go and see a dentist when I feel pain	176	46
	Go and see a dentist immediately	119	31.1
Tobacco causes oral cancer	Yes	228	59.5
	No	155	40.5
Sweet foods affect teeth adversely	Yes	322	84.1
	No	61	15.9
Knowledge of interdental aids	Yes	133	34.7
	No	250	65.3

**Table 4:** Factors associated with oral hygiene practices among patients in dental clinics, Hawassa City Administration, Southern Ethiopia, 2018 (*n* = 383).

Variables	Category	Practice		COR (95% CI)	AOR (95% CI)
		Poor	Good		
Sex	Male	92	112	1.59 (1.05–2.40)	1.63 (1.053, 2.523)^*∗*^
	Female	61	118	1.00	1.00
Age in years	<18 years	9	15	0.89 (0.382, 2.102)	0.722 (0.277, 1.880)
	≥18 years	144	215	1.00	1.00
Educational level	Below secondary	26	22	1.94 (1.053, 3.559)	1.77 (0.887, 3.543)
	Secondary and above	127	208	1.00	1.00
Average monthly household income in ETB	<5000	77	97	1.39 (0.921, 2.095)	1.13 (0.724, 1.749)
	≥5000	76	133	1.00	1.00
Employed	Yes	82	136	1.00	1.00
	No	9	22	1.25 (0.829, 1.892)	1.266 (0.797, 2.011)
Residence	Rural	28	10	4.93 (2.317, 10.481)	3.79 (1.724, 8.317)^*∗∗*^
	Urban	125	220	1.00	1.00
Knowledge	Poor	27	77	2.35 (1.428, 3.863)	2.38 (1.402, 4.024)^*∗∗*^
	Good	126	153	1.00	1.00

^*∗*^ = significant at *P* value <0.05; ^*∗∗*^ = significant at *P* value = 0.001; COR = crude odds ratio; CI = confidence interval; AOR = adjusted odds ratio.

## Data Availability

The data used to support the findings of this study are available from the corresponding author upon request.
